# Rare HBV Genotypes and Clinically Relevant Mutations of HBV and HCV During the COVID-19 Pandemic in a National Reference Outpatient Clinic in Rio de Janeiro, Brazil

**DOI:** 10.3390/v18080872

**Published:** 2026-08-10

**Authors:** Lucas Lima da Silva, Bárbara Vieira do Lago, Vanessa Duarte da Costa, Viviane Brandão Gomes de Sousa, Lia Laura Lewis-Ximenez, Vanessa Salete de Paula, Livia Melo Villar

**Affiliations:** 1 Brazilian Reference Laboratory for Viral Hepatitis, Institute Oswaldo Cruz, Fiocruz, Rio de Janeiro 21040-900, Brazil; barbaravlago@gmail.com (B.V.d.L.); v.duarte391@gmail.com (V.D.d.C.); vivianebrandaogs@gmail.com (V.B.G.d.S.); lialewis.fiocruz@gmail.com (L.L.L.-X.); liviafiocruz@gmail.com (L.M.V.); 2 Molecular Virology and Parasitology Laboratory, Institute Oswaldo Cruz, Fiocruz, Rio de Janeiro 21040-900, Brazil; vdepaula.fiocruz@gmail.com

**Keywords:** viral hepatitis, HBV, HCV, genotypes, COVID-19, Brazil, antiviral resistance, mutations

## Abstract

Chronic hepatitis B and C are major causes of cirrhosis, hepatocellular carcinoma, and liver-related mortality worldwide. The genetic diversity of hepatitis B virus (HBV) and hepatitis C virus (HCV) influences disease progression, diagnosis, vaccine response, and antiviral treatment. Periods of healthcare disruption, such as the COVID-19 pandemic, reinforce the importance of molecular surveillance to monitor viral genotypes and clinically relevant mutations. This study described the distribution of HBV and HCV genotypes and mutations in Rio de Janeiro, Brazil, during the COVID-19 pandemic. A cross-sectional study included 25 patients (15 HBV and 10 HCV) recruited between 2020 and 2022. Viral nucleic acids were amplified and sequenced by Sanger methodology, and mutations were analyzed using the Geno2pheno platform. HBV genotype A predominated (67%), comprising subgenotypes A1 (40%) and A2 (27%), followed by the rare genotypes B1 (13%), F2 (13%), and G (7%). Among HCV-infected individuals, genotypes 1a (40%) and 1b (30%) predominated, followed by genotypes 4 (20%) and 2 (10%). HBV immune escape mutations (Y100C and T126S) and unusual insertions in the S and RT domains were identified. In HCV, the substitutions C316Y, C316N, and S282R were detected. These findings highlight the importance of molecular surveillance for detecting clinically relevant viral variants.

## 1. Introduction

Viral hepatitis is a disease with a major impact on world health, especially hepatitis B and C viruses, with a high level of chronification and mortality, and an estimated total of more than 354 million infected worldwide. Latest reports from the World Health Organization (WHO) on global hepatitis reveal that hepatitis-related deaths have risen to 1.3 million and, every day, more than three thousand people are dying from viral hepatitis. Of this number, hepatitis B and C lead to 83% and 17% of these deaths, respectively [1].

Although the main characteristic of these infections is liver damage, they can differ in terms of viral genetic aspects, transmission mode and geographical distribution [2]. To combat viral hepatitis B and C as a threat to public health, the WHO has set a 2030-year deadline for the elimination of viral hepatitis, based on a multi-professional effort to diagnose, monitor, treat and cure individuals through epidemiological investigations [3].

Brazil remains a country with a substantial burden of chronic hepatitis B virus (HBV) and hepatitis C virus (HCV) infections despite significant advances in prevention, diagnosis, and treatment. According to the latest Brazilian Ministry of Health Epidemiological Report, 302,351 confirmed HBV cases and 342,328 confirmed HCV cases were reported between 2000 and 2024, with HCV accounting for the largest proportion of chronic viral hepatitis notifications nationwide [4]. The epidemiology of viral hepatitis is markedly heterogeneous across the country, with HBV cases concentrated mainly in the Southeast and South regions, whereas HCV is predominantly reported in the Southeast. These regional differences likely reflect variations in population demographics, transmission patterns, healthcare access, and surveillance capacity [4]. This heterogeneous epidemiological scenario underscores the need for sustained surveillance strategies, including molecular characterization of circulating viral genotypes and clinically relevant mutations, to support timely diagnosis, guide public health interventions, and strengthen progress toward the World Health Organization goal of eliminating viral hepatitis as a public health threat by 2030 [3]. However, the COVID-19 pandemic has directly affected the care and monitoring of patients infected with viral hepatitis, as well as clinical and laboratory screening to identify cases, which may impact the goal of eliminating viral hepatitis by 2030 [5,6]. As a result, delayed identification of these individuals may occur at more advanced stages of the liver disease [5].

In Brazil, hepatitis B vaccination represents the cornerstone of HBV prevention. Universal infant vaccination was progressively implemented through the National Immunization Program (PNI) during the 1990s and has been recommended nationwide since 1998. In 2016, vaccine eligibility was expanded to all age groups regardless of risk factors, further strengthening HBV prevention and contributing to the reduction in HBV transmission and its long-term complications [7]. Similarly, direct-acting antivirals (DAAs) were incorporated into the Brazilian Unified Health System (SUS) in 2015, representing a major milestone in hepatitis C therapy. The introduction of highly effective, interferon-free regimens transformed the clinical management of chronic HCV infection by substantially improving sustained virological response rates and expanding access to treatment [8,9]. According to the current Brazilian Clinical Protocol and Therapeutic Guidelines, chronic HBV infection is primarily treated with high genetic barrier nucleos(t)ide analogs, including entecavir and tenofovir-based regimens, whereas chronic HCV infection is managed with pangenotypic direct-acting antiviral regimens, mainly sofosbuvir/velpatasvir or glecaprevir/pibrentasvir, which have replaced genotype-specific therapies and achieve sustained virological response rates exceeding 95% in most patients [10,11].

Nevertheless, the identification of viral genotypes and clinically relevant mutations remains fundamental to effective strategies against viral hepatitis. In addition to enabling epidemiological characterization of geographical distribution, genotypic analysis provides important prognostic information, since specific genotypes are associated with more aggressive disease courses and may require intensified clinical monitoring and therapeutic optimization [12,13,14]. Moreover, the compact and highly overlapping genomic organization of HBV means that a single nucleotide substitution may simultaneously affect multiple viral proteins, particularly the polymerase and surface genes, thereby contributing to both antiviral resistance and immune escape [15]. Furthermore, genotypic characterization allows the identification of resistance-associated substitutions (RASs), which may reduce susceptibility to specific antiviral agents and influence treatment response in selected clinical scenarios, particularly in cases of treatment failure and retreatment. Although current pan-genotypic DAA regimens for chronic HCV infection achieve sustained virological response rates exceeding 95%, molecular surveillance of HCV RASs remains important for optimizing therapeutic strategies and monitoring the emergence of resistant variants [12,16,17].

HBV is classified into nine genotypes (A–I) and one putative genotype (J) with a genome-wide intergenomic sequence divergence of at least 7.5%. Lago et al. (2024) describe the highest prevalence of genotype A in Brazil (74.3%), followed by D (17.7%), F (7.1%) and E (0.9%) [18]. For hepatitis C, there is a distribution of eight genotypes, classified between numbers 1–8 with 93 identified subgenotypes, with genotype 1 being the most prevalent in Brazil, followed by genotype 3 [19,20]. In fact, Oliveira Correa and Chies, 2024, after analyzing the database, raised the hypothesis that the pandemic affected the distribution of HCV genotypes among global regions, with a difference in the percentage of genotype identification before, during, and after the COVID-19 pandemic [19].The distribution of these viral genotypes has an important factor associated with global geolocation and can trace virological paths of dissemination and disease origins, each genotype being more prevalent in a certain region and in certain population groups [21].

Despite the availability in the literature of studies highlighting the distribution of genotypes, most of the studies defining the prevalence of viral hepatitis in Rio de Janeiro predate 2019, which demonstrates the need for studies investigating the distribution of these genotypes during and after the COVID-19 pandemic [19]. Thus, this study aims to describe the distribution of HBV and HCV genotypes and mutations associated with treatment failure and immune escape during the COVID-19 pandemic in Rio de Janeiro, Brazil, with a focus on investigating if there was a change in genotypic distribution during this unique period in human history and assessing the potential impact of these viral hepatitis mutations on treatment outcomes, diagnosis, and vaccine response, in light of efforts to eliminate viral hepatitis, goals that have already been delayed by the pandemic.

## 2. Materials and Methods

### 2.1. Study Population

This is a cross-sectional study approved by the Research Ethics Committee of the Oswaldo Cruz Foundation (CAAE number 11.177.119.8.0000.5248). The study population was derived from a previously established cohort of 230 individuals recruited between March 2020 and November 2022 at the Viral Hepatitis Outpatient Clinic, Viral Hepatitis Laboratory, Oswaldo Cruz Foundation (Fiocruz), Rio de Janeiro, Brazil, as part of a research project investigating the association between liver disease and COVID-19. Among these participants, 25 individuals had detectable HBV DNA or HCV RNA and were therefore eligible for the present molecular study, including phylogenetic analyses. The distribution of hepatitis among these participants was 15 individuals with hepatitis B (HBV DNA positive) and 10 with hepatitis C (HCV RNA positive).

The inclusion criteria were age over 18 years, detectable HBV DNA or HCV RNA, and regular follow-up at the outpatient clinic. Exclusion criteria comprised age < 18 years, absence of detectable viral load, inability to obtain biological samples due to difficult venous access (including patients undergoing hemodialysis), pregnancy, presence of cognitive or mental health conditions that impaired the participant’s ability to provide informed consent or complete the questionnaire, and refusal or inability to sign the informed consent form. All eligible participants completed a structured questionnaire to collect sociodemographic and clinical data. The questionnaire applied in this study was previously developed and published by our group and was used without modifications [22].

### 2.2. Molecular Tests for Viral Hepatitis

Viral DNA and RNA were extracted using the QIAamp^®^ DNA Blood Mini Kit (QIAGEN, Hilden, Germany) and QIAamp^®^ Viral RNA Mini Kit (QIAGEN, Hilden, Germany), respectively, and according to the manufacturer’s protocol for serum samples, obtained by collecting peripheral venous blood and stored in aliquots at −20 °C. The HBV PCR amplified a fragment spanning nucleotides 204–1099 of the overlapping S/Pol region (~900 nucleotides), with nucleotide numbering based on the EcoRI restriction site, designated as position 1 of the 3221 bp genotype A reference genome. The sequences of the primers used for PCR amplification and sequencing were described previously [23]. The HCV PCR amplified nucleotides 8278–8636 (~370 nucleotides) of the NS5B region, with nucleotide numbering according to a previously described reference sequence [24]. PCR amplification and sequencing were performed as previously described [25].

Once amplification was complete, it was confirmed using 1.5% agarose gel electrophoresis. The amplified products were purified, quantified and subjected to the sequencing reaction using the commercial reagent “BigDye™ Terminator v3.1 Cycle Sequencing Kit” (Thermo Fisher Scientific, Waltham, MA, USA). After the in-house precipitation step of the sequencing reaction, the run took place on the 3500 Genetic Analyzer sequencer (Thermo Fisher Scientific, Waltham, MA, USA). Viral genomic regions were amplified using previously validated primer sets. For HBV, an approximately 1 kb fragment spanning the overlapping surface and polymerase (S/Pol) genes was amplified using primers HBV1F (5′-TAGGACCCCTGCTCGTGTTACAGG-3′) and HBV4R (5′-GAAAGGCCTTGTAAGTTGGCG-3′), as described by Mallory et al. Bidirectional Sanger sequencing was performed using the amplification primers (HBV1F and HBV4R) together with the internal sequencing primers HBV2F (5′-CATCCTGCTGCTATGCCTCATC-3′) and HBV3R (5′-GATGGGATGGGAATACAGGTGC-3′), generating approximately 895 bp of a high-quality sequence spanning the S/Pol region [23].

For HCV, a partial NS5B fragment (~382–401 bp), corresponding approximately to nucleotides 8245–8645 of the H77 reference genome (GenBank accession M62321), was amplified using the primers Pr1 (5′-TGGGGATCCCGTATGATACCCGCTGCTTTGA-3′), Pr2 (5′-GGCGGAATTCCTGGTCATAGCCTCCGTGAA-3′), Pr3 (5′-TATGAYACCCGCTGYTTTGACTC-3′), Pr4 (5′-GCNGARTAYCTVGTCATAGCCTC-3′), and Pr5 (5′-GCTAGTCATAGCCTCCGT-3′), according to Sandres-Sauné et al. Bidirectional Sanger sequencing was performed using the corresponding PCR primers [25].

The nucleotide sequences obtained (sense and reverse complementary) from each sample were edited and assembled in the MEGA X software (version X; Temple University, Philadelphia, PA, USA) to obtain the consensus. The evolutionary relationships between the study sequences and the reference sequences representing the HBV and HCV genotypes retrieved from GenBank were determined by phylogenetic tree analysis. The accession numbers of the reference sequences are shown in the phylogenetic tree. The phylogenies were reconstructed by the maximum likelihood (ML) method using the General Time Reversible + Gamma + Invariable sites (GTR + I + G) model of nucleotide substitution, which was selected as the most suitable model according to Akaike’s information criterion in the jModelTest version 2.1.10 (University of Vigo, Vigo, Spain) (PMID: 22847109). The phylogenetic tree was made using the maximum likelihood method in the MEGA X program with a bootstrap resampling of 1000 replicates. Resistance-associated mutations were characterized using the online tool Geno2pheno [hbv/hcv] (Max Planck Institute for Informatics, Saarbrücken, Germany), which analyzes viral sequences to predict potential clinical impact based on known resistance-associated mutations [26].

## 3. Results

Table 1 presents the sociodemographic characteristics of the study participants. The median age was 42 years (IQR, 33–53) among individuals with hepatitis B and 56.5 years (IQR, 44–59) among those with hepatitis C, indicating that most participants were in the fourth to sixth decades of life. There was a similar gender distribution among the total population, with a higher prevalence of women of (56%, 14–25). Regarding self-reported race/ethnicity, most individuals self-identified as Black (36%; 9–25), but self-declaration of Asian origin was found in two (13%) patients with hepatitis B. Most of the individuals had completed high school (56%) or higher graduation and the majority live in the city of Rio de Janeiro (88%).

The distribution of clinically relevant mutations and insertions among hepatitis B virus sequences is presented in Table 2. It was observed that two sequences had the Y100C mutation, and one had a T126S mutation, both in the S domain; these mutations were identified in the HBV/A1. Regarding insertions, two sequences (HBV/G and HBV/F2) harbored a 91-aa insertion in the S domain, which corresponded to a 116-aa insertion in the reverse transcriptase (RT) domain of the overlapping polymerase gene.

**Figure 1:**
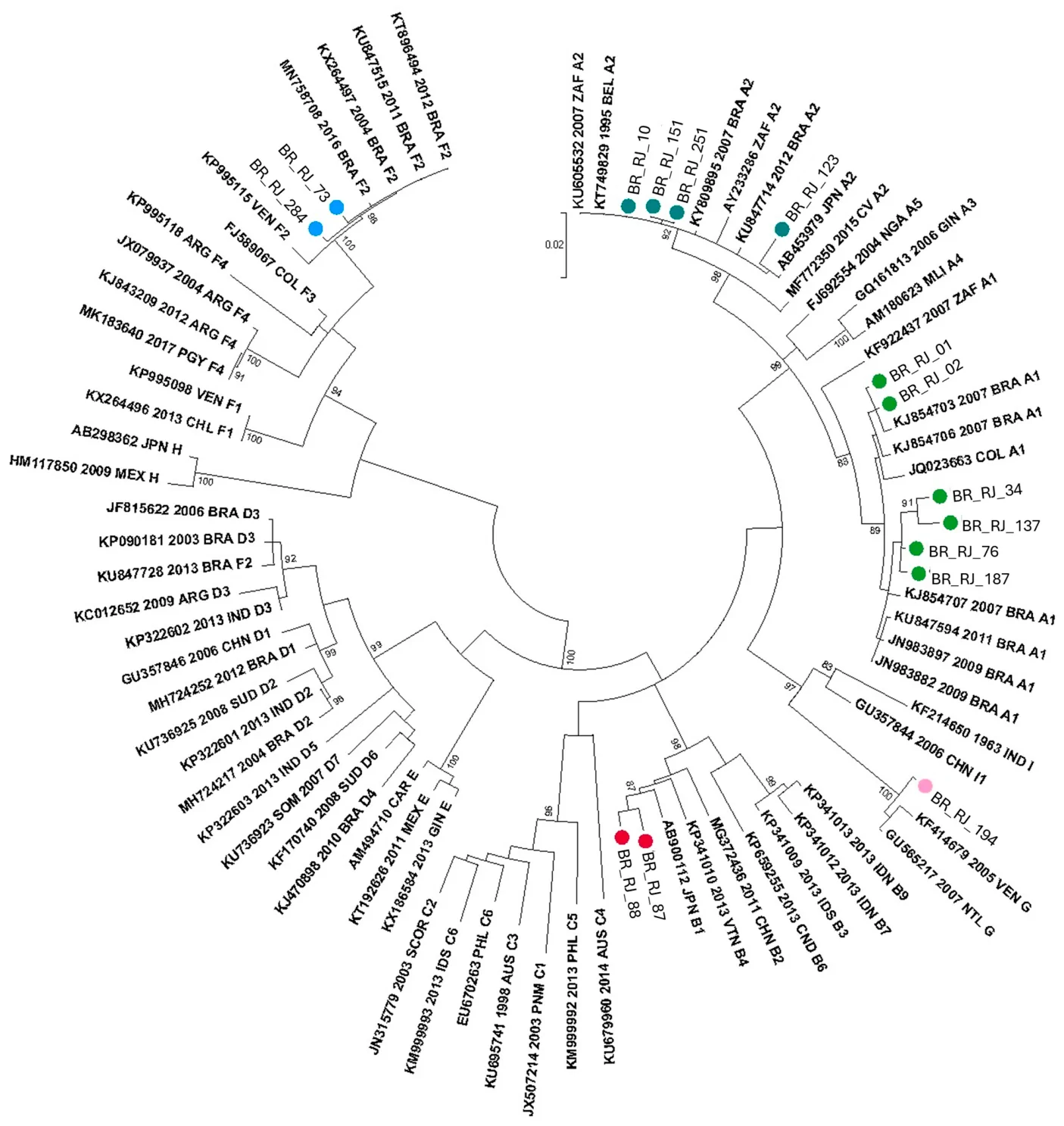
Maximum likelihood phylogenetic tree for genotypic analysis of hepatitis B virus (*n* = 15). The phylogenetic tree was constructed using the maximum likelihood method in MEGA X and included 52 reference sequences. Branch support was assessed by bootstrap analysis with 1000 bootstrap replicates. The colors represent genotype/subtype clades: green, A1; dark blue, A2; light blue, F2; red, B1; and pink, G.

According to the phylogenetic analysis (Figure 1), HBV genotype A1 was the most prevalent 40% (6/15), followed by A2 in 27% (4/15), B1 and F2 in 13% each (2/15) and one individual (7%) carrying the genotype G.

**Figure 2:**
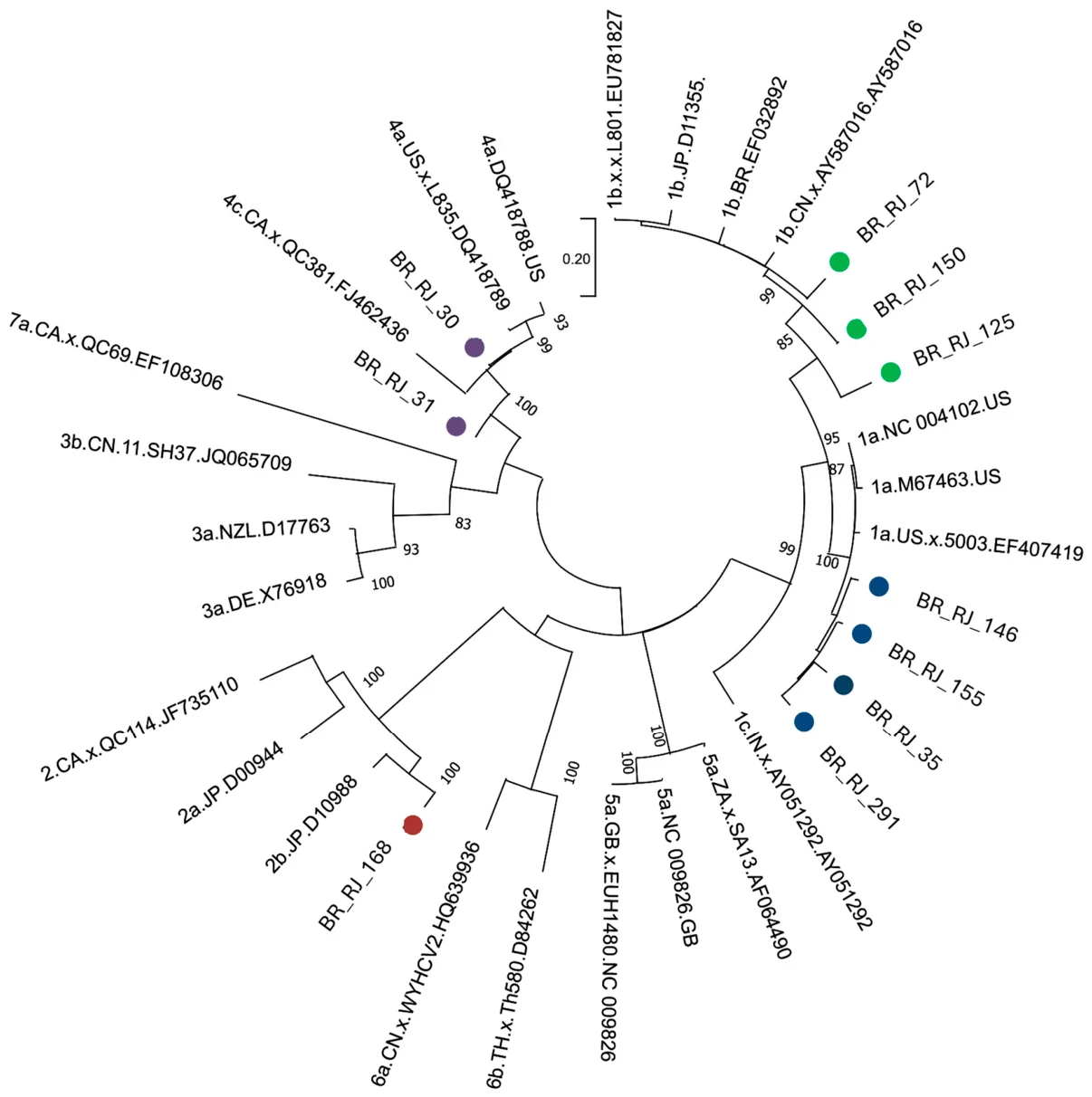
Maximum likelihood phylogenetic tree for genotypic analysis of hepatitis C virus (*n* = 10). The phylogenetic tree was constructed using the maximum likelihood method in MEGA X and included 22 reference sequences. Branch support was assessed by bootstrap analysis with 1000 bootstrap replicates. The colors represent genotype/subtype clades: blue, 1a; green, 1b; purple, 4; and dark red, 2.

Figure 2 illustrates the distribution of genotypes in relation to hepatitis C. Genotype 1A was the most prevalent among the individuals, with 40% (4/10), followed by genotype 1B, with 30% (3/10). Genotype 4 was observed in two individuals (20%) and one individual had genotype 2 (10%).

Mutations associated with or related to HCV resistance are shown in Table 3. One confirmed resistance mutation (C316Y) was identified in one sequence, while two potentially susceptible mutations (C316N and S282R) were detected individually, each in a distinctive sequence.

## 4. Discussion

The distribution of hepatitis B genotypes found in this study represents the distribution of genotypes in Brazil, with genotype A being the most prevalent; HBV/A1 (79.7%) and HBV/A2 (20.3%) according to the study by Lago et al. (2024) [18]. These data are consistent with previous studies showing that subgenotypes A1 and A2 are widespread in Brazil and across the Americas [27,28,29]. Studies demonstrate that subgenotype HBV/A1 is the most prevalent in the city of Rio de Janeiro [30,31].The origin of subtype A1 has been associated with the African diaspora and A2 with European migration [28]. The distribution of these genotypes in Brazil reveals aspects of the country’s population history. In our study, two genotypes that are rarer in Brazil were found. HBV/B is characteristic of Asia, while HBV/G is a rare genotype found in restricted regions of the world [28].

The rareness of HBV/B in Brazil is highlighted in a multicenter study developed with 1000 samples from all geographic regions of Brazil, where only one sample was observed for HBV/B (0.1%) [27]. There are few reports of this genotype being identified in Brazil. This is a genotype characteristic of East Asia and may be associated with the migratory profile of foreigners to Brazil [18,27]. Correlating the characteristics of the viral genotype, the two individuals carrying HBV/B in our study are of Asian origin, representing the only ones who self-declared themselves as having yellow skin color. Both are direct relatives, being sisters, which reinforces the hypothesis of intrafamilial transmission. The presence of genotype B, considered rare and restricted to Asian countries, illustrates how the genetic diversity of HBV can reflect historical migratory routes and specific ethnic links. This finding also highlights the importance of including sociodemographic and genetic data for more accurate epidemiological tracking of viral infections.

Another rare genotype found in our study was HBV/G (7%–1/15), often associated not only with a specific geographical distribution but also with specific population groups [32]. This is a genotype with low global identification and whose origin and routes of dissemination are still poorly understood [21]. Similar percentages to our study were observed in other studies, in a work that evaluated the prevalence of hepatitis among men who have sex with men in 12 Brazilian cities; this genotype was the least prevalent, in 5.6% of samples [32]. Another study conducted in Rio de Janeiro and Mato Grosso states, three individuals (8.3%–3/36) were found to be carriers of isolated HBV/G [13]. Due to its unique molecular features—including a 36–base pair (bp) insertion in the core gene and two stop codons in the precore (pre-C) region—reports of genotype G monoinfection, as observed in our study, are rare. Although the sequencing strategy employed does not allow inference regarding the presence or absence of coinfection with another genotype, this finding highlights the uniqueness of our cohort of individuals with hepatitis B [33].The HBV genotype F, identified in 13% of the individuals in our study, is considered endemic in regions of the Americas, especially in South America, and is associated with indigenous populations and historical contexts of migration [14,18]. A prevalence of 13% was also found in a Brazilian study that investigated blood donors [14].

Two mutations related to immune escape (Y100C and T126S) were identified among HBV sequences, all in genotype A1. These mutations are associated with the absence of HBsAg detection and hepatitis B vaccine escape [34]. In a scenario aggravated by the COVID-19 pandemic, with reduced diagnosis of viral hepatitis, with HBsAg being used as a diagnostic marker through rapid tests, and growing vaccine hesitancy, the identification of these variants is particularly concerning, as it highlights the circulation of strains potentially not neutralized by the immune and vaccine response [6,35]. Reduced vaccination coverage may favor the spread of these mutants, compromising advances in hepatitis B control and posing an additional risk to public health. In two samples, one of genotype G and the other of F2, unusual insertions were identified in critical regions of the HBV genome. Due to the overlapping nature of the HBV genome, the insertion of a single nucleotide may affect multiple viral proteins. In this case, the p.91_92insA insertion identified in the *S* gene, which introduces a lysine residue, corresponds to the p.116_117insA insertion within the RT domain of the polymerase gene. Consequently, this insertion leads to the addition of a glutamate residue followed by a premature stop codon at position 118 of the RT domain. Although this mutation would be expected to abolish polymerase activity, viral replication may nevertheless be sustained by viral subpopulations harboring an intact polymerase.

Furthermore, although these insertions have been rarely reported and remain poorly characterized, previous studies suggest that insertions affecting the antigenic structure of HBsAg may compromise recognition by diagnostic assays and alter immune recognition [36,37]. Regarding genotype A1 sequences, although no HBV drug resistance-associated mutations were identified, the detection of immune escape-associated substitutions in the SHB region (Y100C and T126S) is noteworthy, given their potential to impair HBsAg recognition by diagnostic assays and contribute to hepatitis B vaccine escape.

The distribution of HCV genotypes found in our studies is consistent with the Brazilian distribution [38]. Here, genotypes 1b and 1a were the most prevalent, followed by 4 and 2. Noteworthy is the absence of genotype 3 in our cohort, the second most prevalent genotype in Brazil [39]. One hypothesis was raised in a previous study [19], wich evaluated the distribution of HCV genotypes before, during, and after the COVID-19 pandemic, and found that pandemic factors such as reduced mobility and reduced screening can alter the effective circulation of viruses in populations and, consequently, the relative frequency of circulating genotypes, while raising indirect signs in the literature such as the emergence of a new subtype of HCV genotype 4 recently identified in Saudi Arabia.

RAS C316Y was observed in an individual carrying genotype 1A. This mutation confers resistance to dasabuvir, used in Brazil in combination with other direct-acting antivirals in severe cases of liver damage and cirrhosis associated with hepatitis C, which draws attention to this mutation for possibly compromising therapeutic efficacy in critically ill patients indicated for this treatment, especially when pan-genotypic therapeutic alternatives are not available or are contraindicated [11].

The substitutions susceptible to resident S282R and C316N in the NS5B region of HCV found in two individuals with genotype 1b are associated with resistance to direct-acting antivirals. S282R has been described in association with possible resistance to sofosbuvir, a nucleoside inhibitor of NS5B widely used in the treatment of viral hepatitis C and considered clinically relevant, since it can compromise the effectiveness of treatment, a rare situation when considering a drug as effective and revolutionary as sofosbuvir [39]. The C316N substitution is related to resistance to dasabuvir, a non-nucleotide NS5B inhibitor, with moderate impact, especially in individuals infected with genotype 1b, such as the carriers in our study [40]. Both can arise under selective pressure and should be considered when choosing a treatment regimen, even in current therapeutic regimens that use combinations of multiple antivirals.

Clinically relevant resistance-associated mutations in HCV are primarily evaluated in the NS3, NS5A, and NS5B regions, depending on the direct-acting antiviral (DAA) regimen and the clinical context. Although current first-line DAA regimens are predominantly pan-genotypic and achieve sustained virological response rates exceeding 95% across HCV genotypes, thereby reducing the need for routine baseline resistance testing, the characterization of resistance-associated mutations remains clinically relevant. Inzaule et al. (2024) recently demonstrated that, despite the low rate of virological failure with pan-genotypic regimens, 79–96% of patients experiencing treatment failure harbor at least one resistance-associated substitution, highlighting the importance of continued resistance surveillance to optimize retreatment strategies [41]. Likewise, Nunes et al. (2026) reported that resistance-associated substitutions are heterogeneously distributed across HCV genotypes and geographic regions, with persistent NS5A-associated substitutions in the pan-genotypic DAA era, whereas sofosbuvir retains a high genetic barrier to resistance [42]. Together, these findings reinforce the importance of ongoing molecular surveillance to monitor emerging resistance patterns and support optimized antiviral therapy and HCV elimination strategies.

Although the resistance-associated mutations identified in this study were detected exclusively through sequence analysis and were not linked to documented treatment failure, their characterization remains relevant for understanding the molecular epidemiology and genetic diversity of circulating HBV and HCV strains. These findings provide a baseline for regional resistance surveillance and may help identify mutations with potential clinical significance as antiviral treatment strategies continue to evolve.

The sociodemographic data of the individuals are consistent with data in the literature regarding the age of follow-up of patients with viral hepatitis from the fourth decade of life, due the chronic nature of the disease [2]. The higher concentration of women is largely associated with the fact that women access basic health services more than men [43].

This study has some limitations that should be acknowledged. The resistance-associated mutations identified were detected exclusively by sequence analysis, and no clinical follow-up or treatment outcome data were available to determine whether these variants were associated with antiviral treatment failure. Moreover, although all samples were collected before the initiation of antiviral therapy, information on subsequent antiviral treatment and therapeutic outcomes was unavailable, precluding the evaluation of the clinical impact of the identified mutations. Therefore, the identified mutations should be interpreted as molecular findings with potential clinical relevance rather than confirmed predictors of therapeutic response. Nevertheless, these limitations do not diminish the importance of the present findings. Characterizing resistance-associated mutations contributes to understanding the genetic diversity of circulating HBV and HCV strains, supports molecular epidemiological surveillance, and provides baseline data for future studies integrating genomic, clinical, and therapeutic outcome information. Such surveillance remains relevant even in the era of highly effective pan-genotypic antiviral therapies, particularly for monitoring emerging resistant variants and informing retreatment strategies.

## 5. Conclusions

This study identified the predominance of HBV/A and HCV/1a genotypes during the COVID-19 pandemic in Rio de Janeiro, with the presence of rare genotypes such as HBV/B and HBV/G in Brazil. Additionally, mutations related to immune escape in HBV and resistance-associated substitutions in HCV NS5B were detected, suggesting that disruptions in healthcare access and surveillance during the COVID-19 pandemic may have influenced the circulation, detection, and persistence of clinically relevant viral variants, highlighting potential challenges for diagnosis, vaccine efficacy, and antiviral treatment.

## Figures and Tables

**Table 1:** Sociodemographic characteristics and liver biochemical markers of individuals with viral genotypes sequenced for the study (*N* = 25).

Groups	Hepatitis B (*n* = 15)	Hepatitis C (*n* = 10)	Total (*N* = 25)
Characteristics			
Age, years, median (IQR)	42 (33–53)	56.5 (44–59)	46 (35.5–58.5)
Gender (%)			
Female	7 (47%)	7 (70%)	14 (56%)
Male	8 (53%)	3 (30%)	11 (44%)
Race/Ethnicity			
Black	6 (40%)	3 (30%)	9 (36%)
Brown	6 (40%)	2 (20%)	8 (32%)
White	1 (7%)	5 (50%)	6 (24%)
Asian	2 (13%)	-	2 (8%)
Education level (%)			
≥high school	9 (60%)	5 (50%)	14 (56%)
≤elementary school	6 (40%)	5 (50%)	11 (44%)
City of residence (%)			
City of Rio de Janeiro	15 (100%)	7 (70%)	22 (88%)
Other city in the state of Rio de Janeiro	-	3 (30%)	3 (12%)

**Table 2:** Distribution of clinically relevant HBV mutations observed in the study (*n* = 15).

HBV Genotype	SHB Protein Mutations Associated with Immune Escape *n* (%)	
A1	T126S	1 (6.6%)
A1	Y100C	2 (13.3%)
	Insertion in the SHB protein domain	
G/F2	p.91_92insA	2 (13.3%)
	Insertion in the RT domain	
G/F2	p.116_117insA	2 (13.3%)

SHB: small hepatitis B surface protein; RT: reverse transcriptase.

**Table 3:** Distribution of clinically relevant HCV mutations observed in the study (*n* = 10).

HCV Genotypes	NS5B Substitutions Associated with Resistance (RAS) *n* (%)	
1A	C316Y	1 (10%)
	NS5B Susceptible substitution	
1B	C316N	1 (10%)
1B	S282R	1 (10%)

## Data Availability

The nucleotide sequences generated and analyzed during the current study are available in the GenBank repository under accession numbers PZ387075–PZ387089 for HBV sequences and PZ387105–PZ387114 for HCV sequences.

## Abbreviations

The following abbreviations are used in this manuscript:

bp	Base pairs
CAAE	Certificate of Presentation for Ethical Consideration
CAPES	Coordination for the Improvement of Higher Education Personnel
CEP	Research Ethics Committee
COVID-19	Coronavirus Disease 2019
DAA	Direct-acting antivirals
Fiocruz	Oswaldo Cruz Foundation
GTR + I + G	General Time Reversible model with Invariable sites and Gamma distribution
HBsAg	Hepatitis B surface antigen
HBV	Hepatitis B virus
HCV	Hepatitis C virus
ML	Maximum likelihood
NS5B	Non-structural protein 5B
PCR	Polymerase chain reaction
RAS	Resistance-associated substitutions
RT	Reverse transcriptase
SD	Standard deviation
SHB	Small hepatitis B surface protein
WHO	World Health Organization
